# Hierarchical Self-Assembly of Proteins Through Rationally Designed Supramolecular Interfaces

**DOI:** 10.3389/fbioe.2020.00295

**Published:** 2020-04-21

**Authors:** Hongcheng Sun, Yan Li, Shuangjiang Yu, Junqiu Liu

**Affiliations:** ^1^College of Material, Chemistry and Chemical Engineering, Hangzhou Normal University, Hangzhou, China; ^2^Key Laboratory of Organosilicon Chemistry and Material Technology, Ministry of Education, Hangzhou Normal University, Hangzhou, China

**Keywords:** protein self-assembly, protein-protein interactions, supramolecular interfaces, hierarchical nanostructures, biofunctionalization

## Abstract

With the increasing advances in the basic understanding of pathogenesis mechanism and fabrication of advanced biological materials, protein nanomaterials are being developed for their potential bioengineering research and biomedical applications. Among different fabrication strategies, supramolecular self-assembly provides a versatile approach to construct hierarchical nanostructures from polyhedral cages, filaments, tubules, monolayer sheets to even cubic crystals through rationally designed supramolecular interfaces. In this mini review, we will briefly recall recent progress in reconstituting protein interfaces for hierarchical self-assembly and classify by the types of designed protein-protein interactions into receptor-ligand recognition, electrostatic interaction, metal coordination, and non-specific interaction networks. Moreover, some attempts on functionalization of protein superstructures for bioengineering and/or biomedical applications are also shortly discussed. We believe this mini review will outline the stream of hierarchical self-assembly of proteins through rationally designed supramolecular interfaces, which would open minds in visualizing protein-protein recognition and assembly in living cells and organisms, and even constructing multifarious functional bionanomaterials.

## Why Protein Self-Assembly

Proteins are most important functional players that implement difficult but essential tasks in living cells. However, most proteins in nature execute their biological missions in the form of protein clusters through self-assembly with sophisticated topological structures and versatile functionalities, such as tubular α-hemolysin (Tanaka et al., 2011), helical tobacco mosaic virus (TMV) (Ge and Zhou, 2011), polyhedral carboxysome (Tanaka et al., 2008), and well differentiated bacteriophage T4 (Leiman et al., 2003). It is suspected that proteins can spontaneously go to assembled superstructures with well-organized quaternary structures. In this process, protein–protein interactions are the chief contributors for the diversified protein nanostructures, mediated by the protein orientation (Luo et al., 2016; Nguyen et al., 2019). How to design a pair of complementary supramolecular interfaces will help to construct hierarchical protein nanostructures *via* supramolecular self-assembly.

Protein self-assembly is the predominant means of building complexity in living systems. The following two aspects must be clear before starting with protein self-assembly: supramolecular interaction and protein symmetry. Various kind of supramolecular interactions are involved in protein assemblies, such as hydrophobic interactions, amphiphilic interfaces, hydrogen bond networks, *Van der Waals* interactions, receptor-ligand recognition, and metal coordination and so on (Bai et al., 2016). These driving forces have yielded both discrete and infinite/periodic assemblies which exhibit dynamic behavior and novel mechanical attributes. With fully considering the structural symmetry of proteins, such supramolecular interactions can be employed to construct more complicated protein superstructures including but not limited to polyhedral cages, fibrils, rings, tubules, planar sheets, or even macroscopic superlattices (Sun et al., 2017). Also, the structural, functional and mechanical properties of such protein nanostructures are far beyond those explored by natural evolution. However, it is urgently needed to be addressed in respect of formation mechanisms and new opportunities in the next period. For example, how to design the supramolecular protein interface to predict self-assembly superstructures. Such knowledge will facilitate the development of general protocols for self-assembly of proteins and further for developing defined nanomaterials for biomimetics or biomedical applications.

This tutorial review paper stresses the importance of interfacial interactions and structural symmetry in guiding the self-association of protein building blocks, and further constructing hierarchical and multidimensional superstructures. In addition, the constructed hierarchical structures are potentially promising templates for development of bioinspired materials for catalysis, sensing, and environmental or biomedical applications.

## Toolsets From Designed Supramolecular Interfaces

The spirit of hierarchically constructing protein assembly is how to design the supramolecular protein interfaces. By employing the protein docking technique, proteins can be assembled directly into protein complex with three-dimensional precise structures as predicted. In this process, the geometrical symmetry and the interfacial bonding position of building blocks determine the topological network structure of complex. And the supramolecular bonding mode can also affect the structural stability and responsibility. Besides, most natural proteins exhibit weak protein-protein interactions and quite easily misrecognizing to random aggregates. Therefore, protein interfaces are generally reconstructed from native proteins to realize high specificity and selectivity *via* gene mutation, protein fusion, chemical modification, etc., which are difficult to design from scratch. Up to now, numerous kinds of proteins, such as cytochrome, cowpea chlorotic mottle viruses (CCMV), lectins, stable protein one (SP1), glutathione S-transferases (GSTs), chaperonin GroEL, etc., have been employed and showed great potential in developing different protein topological structures with advanced functional properties.

**FIGURE 1:**
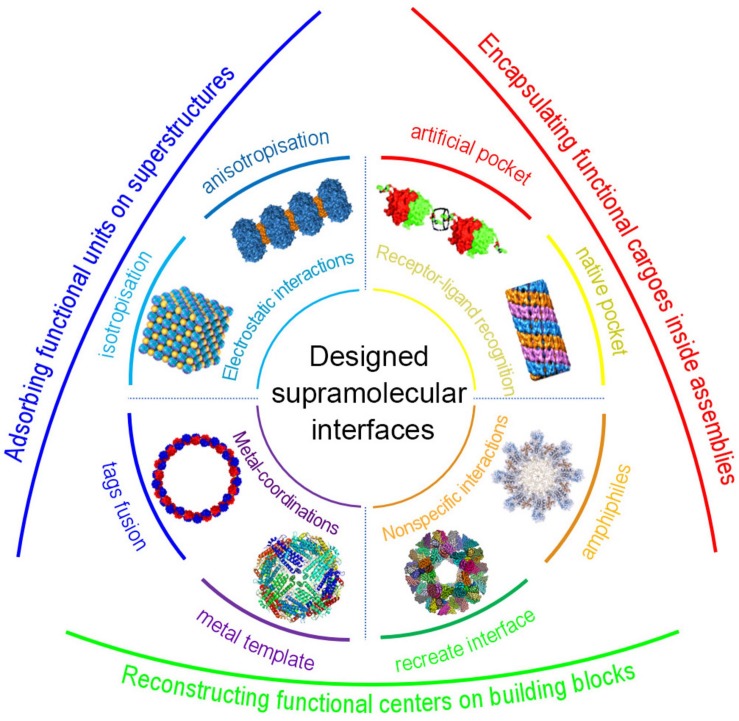
Schematic representation of protein self-assembly through designed supramolecular interactions and their biofunctionalization. Receptor-ligand recognition with native pocket and artificial pocket, reproduced with permission from Hou et al. (2013) and Li X.M. et al. (2019), respectively; Electrostatic interactions into anisotropic and isotropic structures, reproduced with permission from Sun et al. (2015) and Chakraborti et al. (2019), respectively; Metal-coordination via tags fusion, reproduced with permission from Bai et al. (2013), or metal-template-mediated reconstruction; Non-specific interaction networks through recreate interfaces or amphiphiles, reproduced with permission from Xu et al. (2019).

Symmetrical docking is generally the fundamental strategy to artificially construct the hierarchical protein nanostructures. In order to reconstruct the specific low-energy protein interfaces, various kind of supramolecular interactions, such as receptor–ligand recognition, metal coordination, electrostatic interactions, and others non-specific interaction networks, have been successfully employed (Figure 1). Further considering the bond multiplicity and orientation, proteins can be docked into different kinds of spatial orderly frameworks. Herein, we concisely elaborate recent progress depending on the types of the supramolecular driving forces and the controlled morphology (Table 1). We hope this mini review will give colleagues a definite instruction in designing hierarchical protein structures through supramolecular self-assembly strategies.

**TABLE 1:** Representative progresses on protein self-assembly through designed supramolecular interactions.

Proteins	Chaperone	Interfacial interactions	Superstructures	Application fields	References
Heme-decorated cyt *b*562	n.a. or trigeminal hemes	Heme-pocket	Linear or hyperbranched protein necklace	n.a	Kitagishi et al., 2009; Oohora et al., 2018
SAv and dimeric apoMb	heme−bis (biotin)	Heme-pocket and biotin-avidin	Alternating protein nanowires	n.a	Oohora et al., 2012
Lectin (Con A or Lec A)	Rh3Man or Rh3Gal	Sugar-lectin and RhB dimerization	Nanoribbons, nanowires, nanosheets, or superlattices	n.a	Sakai et al., 2014; Yang et al., 2017
Lectin (SBA)	GN3R, M3P, …	Sugar-lectin and dimerization	Helical polymorphism of microtubes	n.a	Li X.M. et al., 2019
FGG-tagged GST	CB[8]	Host-guest	Protein nanowires, nanorings, nanospirals, nanowires, and superwires	Artificial GPx nanoenzyme	Hou et al., 2013; Li et al., 2017
FGG-recoverin-GST fusion	CB[8]	Host-guest	Ca^2+^ responsive dynamic nanospring	n.a	Si et al., 2016
Microtubules	β−CD and AAP	Host-guest	Photo-controlled reversible assembly	Diseases related to improper protein aggregation	Zhang et al., 2018
SP1 protein	QDs, PAMAM, and CCMs	Electrostatic interactions	Programmed protein nanowires	Chloroplast mimics, Multienzyme cascades	Sun et al., 2015, 2016a
TMV	Pc	Electrostatic interactions	Fibrous bundles	light−mediated heterogeneous catalysis	Anaya-Plaza et al., 2019
CCMV and Ferritin	AuNPs, PAMAM, and Avidin	Electrostatic interactions	Protein superlattices with tunable structures	Active enzyme capture and artificial chaperone activity	Chakraborti et al., 2019
His-tagged GST	Ni^2+^ ions	Ni^2+^-His coordination	Protein nanowires or nanorings	n.a	Bai et al., 2013
cyt *b*562 variant	Ni^2+^ ions	Ni^2+^-His coordination	Nanocages, nanotubes, and sheets	n.a	Brodin et al., 2014
Bpy-decorated AAC	Ni^2+^ ions	Ni^2+^-bpy coordination	Protein rods and planes	n.a	Yang and Song, 2019
GroEL_MC_	Mg^2+^ ions	Mg^2+^-MC coordination	High-integrity nanotubes	ATP-regulated drug delivery	Biswas et al., 2013
His-TMV mutant	Cu^2+^ ions	Cu^2+^-His coordination	Highly-porous 2D crystals	Template for inorganic nanoparticle assembly	Zhang et al., 2019
KDPGal/FkpA fusion	n.a	Multiple interfacial interactions	Hexahedral cages	n.a	Lai et al., 2014
AcpS variant	n.a	Multiple interfacial interactions	Dodecameric tetrahedron and tetraicosameric octahedron	n.a	King et al., 2012
Pentamer, trimer or dimer	n.a	Multiple interfacial interactions	Rhombic triacontahedron	Two-component structures	Bale et al., 2016
PNIPAAm-conjugated BSA	n.a	Amphiphilic interfaces	Proteinosomes	Artificial prokaryotic cells	Huang et al., 2013
Polypeptide-conjugated AKe	n.a	Amphiphilic interfaces	Ap5A regulated nanofilaments and rectangular nanosheets	n.a	Xu et al., 2019
					

### Receptor–Ligand Recognition

In living cells or organisms, some native proteins, such as avidin, enzyme, antibody, lectin, etc., generally play critical roles in cellular biochemical pathways to show their physiological effects like “lock and key” model through recognizing substances with extremely strong specific interactions. Such receptor-ligand interactions will offer a great wealth for scientists to construct and modulate *in vitro* protein superstructures. For instance, hemoprotein (cytochrome, hemoglobin) is a conjugate protein who can specifically associate with heme group in its pocket. Through incorporate an external heme colleague at the opposite site of the native heme binding pocket, cytochrome variant (cyt *b*_562_) and myoglobin were able to polymerize to form hemeprotein linear strings (Kitagishi et al., 2007; Oohora et al., 2011, 2018). If introduced with a spot of trigeminal heme ligands, cyt *b*_562_ will automatically assemble into hyperbranched hemeprotein nanowires (Kitagishi et al., 2009). Besides, divalent connector (Biot_2_-terpy), which displayed a metal chelating terpyridine motif and a bis-biotin moiety, would conjugate with streptavidin (SAv) to afford one dimensional metal-organic protein frameworks (Burazerovic et al., 2007). Through covalent coupling two apomyoglobin (apoMb) tail to tail and meanwhile designing a heme-bis(biotin) ligand, a brand-new programmed supramolecular assembly of apoMb and SAv with alternating alignment was developed (Oohora et al., 2012).

Furthermore, receptor–ligand recognition can be employed to construct more sophisticated structures through modulate protein symmetry and ligand conformation. Wagner et al. constructed supramolecular enzyme nanorings utilizing a fused dihydrofolate reductase dimer (DHFR_2_) associated with bivalent enzyme inhibitor methotrexate (MTX_2_-C_9_) (Carlson et al., 2006; Chou et al., 2008). Besides, Chen’s group did great progress in guiding self-assembly of lectins into various structures with different dimensions. By inducing ligands containing monosaccharide and rhodamine groups connected with an oligo(ethylene oxide) spacer (RhMan), the homotetrameric lectin Con A could be directed to three dimensional protein crystalline frameworks (PCFs) with ligand length-controlled interpenetration (Sakai et al., 2014). While using the nearly planar sharped tetrameric soybean agglutinin (SBA) with *D*_2_ symmetry, this strategy could be utilized to develop uniform tubular structure with 9 SBA units for each helix and 19 nm for each screw pitch (Yang et al., 2016). Ligand design through selective modulation of supramolecular interactions could also be utilized to modulate the polymorphism of protein microtubes (Li X.M. et al., 2019). But for another native homotetrameric LecA protein with cuboid sharp, four kind of protein structures from one-dimensional nanoribbons and nanowires, two-dimensional nanosheets, to three-dimensional layered structures were generated by only changing the length of oligo(ethylene oxide) spacer (Yang et al., 2017). These results provide a general platform to construct different protein structures through coupling protein with its native binding pocket.

However, for vast variety of constitutive proteins, they don’t have any specific binding pocket to bind with ligands. Scientists can artificially introduce a specific binding fragment at proper site who can specifically recognize ligands with high affinity, which greatly expanded the application scope of this strategy to develop new protein superstructures (Nguyen et al., 2010). Liu et al. fused two Phe-Gly-Gly (FGG) tripeptides at the shoulder of twofold axisymmetric (C_2_-symmetric) dimeric GST, which could be selectively associated with cucurbit[8]uril (CB[8]) in a 2:1 ratio to string GST up into nanowires (Hou et al., 2013). If FGG tags was fused with a flexible linker and relative “V-shape” orientation, GST was regulated by CB[8] to distinctive morphological diversities ranging from nanorings, nanospirals, nanowires to superwires (Li et al., 2017). Further, fusing FGG tagged recoverin proteins, who can be triggered by Ca^2+^ to conformational change reversibly, to the shoulder of GST dimer, the fusion protein was successfully employed to construct ion signal responsive artificial nano-spring (Si et al., 2016).

### Electrostatic Interaction

Most protein surfaces always afford abundant charge distributions due to the divergent amino acid residues, which might provide an access to guide the self-assembly of proteins with electrostatic interfaces. Developments have been reported by Liu et al. (2016) in one dimensional protein assemblies with electronegative dodecameric SP1 with *C*_6_ symmetrical cricoid structure. They found various positively charged nanoparticles with complementary dimension, such as quantum dots (Miao et al., 2014), dendrimers (Sun et al., 2015), or core-crosslinked micelles (Sun et al., 2016a), could all electrostatically mediate SP1 into supramolecular nanowires through stringing them face to face. Besides, using a photoisomerizable azobenzene-cored poly(amidoamine) dendrimer, they successfully constructed a photocontrolled dynamic protein assemblies with structure reversibly switched between straight nanowires and bending nanorings (Sun et al., 2016b).

Although pioneering developments have been made for synthetic nanoparticles, electrostatic assembly of binary superlattices with protein building blocks remain challenging due to their patchy interfaces. Kostiainen et al. (2013) have made great progress on mechanism study of electrostatic directed protein crystallization with icosahedral CCMV and ferritins (FT). They found the crystallographic arrangement and the superlattice parameters were affected by the dimension ratio and charge distribution. Positively charged AuNPs and PAMAM protein could behave as “interface glue” to guide the assembly of CCMV or FT to periodically arranged superlattices with quite different parameters. AuNPs could guide both CCMV and FT into interpenetrating face-centered cubic (fcc) structure with CCMV–AuNP_8_ (Kostiainen et al., 2013) and FT-AuNP_1_ supralattices (Chakraborti et al., 2019). For similar PAMAM dendrimer, the lattice parameters and structures was tunable with PAMAM-generation dependency from fcc crystals to hexagonal close-packed (hcp) crystals when PAMAM growth from G2 to G7 (Liljestrom et al., 2015). Besides, polypeptide K72 (Korpi et al., 2018), multi-positive dyes (Mikkila et al., 2016), or even patchy avidin (Liljestrom et al., 2014) could all modulate CCMV or FT into three dimensional protein supralattices through electrostatic interactions.

### Metal Coordination

Metal coordination plays essential roles in natural biological systems, including metalloenzymes, chloroplast, and zinc-finger families. The primary challenges for designing supramolecular interfaces are how to definite the specific metal-binding site and stabilize the quaternary superstructures (Sanghamitra and Ueno, 2013). In this topic, Liu et al. (2016) described the proof-of-principle studies for metal-mediated self-assembly of one-dimensional nanofibers based on a *C*_2_-symmetric GST dimer. After introducing two His_6_-tags vertically aligned with the *C*_2_ axis of GST dimers in the opposite direction, His_6_-tagged GST was self-assembly into protein nanofibers mediated by Ni^2+^ (Zhang et al., 2012). This model can be further used to construct more complicated nanorings with accurate control over protein orientation. When two symmetric metal-binding motifs were designed onto the GST surface in a “V-shape” orientation, GST was mediated by Ni^2+^ into ordered protein nanorings through auxiliarily orientation with intrinsic non-specific interactions (Bai et al., 2013).

Metal mediated protein assembly can be also employed for more complicated structures, such as nanotubes, nanosheets, or crystals. Tezcan et al. described the proof-of-principle studies for metal-mediated self-assembly based on a four-helix bundle cyt *cb*_562_ as model protein. Through introducing metal-combinable histidine tags and reconstructing interfacial charges, cyt *b*_562_ variant (RIDC3) could form a stabilized *C*_2_-symmetrical dimer (Zn_2_:RIDC3_2_) through binding Zn^2+^ ion with histidine tags, which could be further guided to assemble into kinetically folded helical nanotubes or stack into thermodynamically stabled two- or three-dimensional crystalline arrays by modulating the nucleation rate (Brodin et al., 2012, 2014). After further engineering a disulfide-linked RIDC3 dimer, the *D*_2_ tetramer (Zn_8_:RIDC3_4_) could be directed by Zn^2+^ ion to assemble into helical protein nanotubes with variable diameters (Brodin et al., 2015). Besides, Yang and Song (2019) genetically incorporated bidentate bipyridine (bpy) into a *D*_3_ homohexameric acetyltransferase (AAC) variants. Depending on the position of metal-chelating bpy, hierarchical architectures with one-directional protein rods and two-directional planes were created through formation of Ni(bpy)_2_ coordination (Yang and Song, 2019). Wang et al. employed larger cylinder-shaped TMV disks to construct well-ordered 2D TMV monolayer sheets *through* metal ions mediated coordination with fused histidine tags on TMV surfaces (Zhang et al., 2019).

Besides, Aida et al. reported a cylindrical chaperonin GroEL protein decorated with photochromic units [spiropyran (SP)/merocyanine (MC)], which could form into long nanotubes in the presence of divalent Mg^2+^ ions (Sim et al., 2015). Interestingly, the assembly and disassembly of the developed GroEL nanotubes could be manipulated by exposure to UV/visible light or biogenic signaling molecule adenosine-5′-triphosphate (ATP) (Biswas et al., 2013). Tezcan et al. employed reverse metal-templated interface redesign (rMeTIR) to transform natural protein-protein interface into one that only binding with metal ion, which found that ferritin variant could be controlled by divalent Cu^2+^ binding into cage-like structures (Huard et al., 2013). In addition, *C*_4_-symmetric l-rhamnulose-1-phosphate aldolase variant (^H63/H98^RhuA) with binary His residues at the vertex of planar quadrate structure can be also mediated by divalent Zn^2+^ or Cu^2+^ ions to yield two-dimensional nanoporous protein lattices (Suzuki et al., 2016).

### Non-specific Interaction Networks

Proteins in organism are always in the form of oligomers to exhibit their functionality through complicated supramolecular networks including hydrogen bonds, *Van der Vaals*, hydrophobic interactions, π-π stacking, and so on. The oligomerization of protein monomer can be employed to synthesize predetermined protein nanostructures directly *in vivo*. For example, native KDPGal generally exists in homotrimeric structure and FkpA in homodimer to stabilize the configuration of themselves. After geometrically engineering each KDPGal and FkpA monomers together with a continuous α-helical linker, the fusion could self-assemble into large polyhedral cages. When monomer orientation on both sides was modulated 36.5° with the linker, the fusion formed nearly perfect 24-meric hexahedral cages with 23 nm outer diameter, although coexisted with little 18-meric triangular prism and 12-meric tetrahedron (Lai et al., 2014). Besides, Arai et al. created a WA20-foldon nanobuilding block by fusing the dimeric WA20 to the trimeric fibritin structure of bacteriophage T4, which could self-assemble to stable oligomeric structures with hexameric barrel and dodecameric tetrahedron (Kobayashi et al., 2015). Meanwhile, this method could be also used for design of more sophisticated 60-mer truncated icosahedral protein cages (TIP60) through fusing pentamer LSm and dimer MyoX-coil with a short linker (Kawakami et al., 2018).

Inspired from nature, rationally engineered protein interfaces could also help to synthesize new protein superstructures *de novo*. Scientists have made great progress in reconstructing low-energy supramolecular interaction networks at the protein interfaces with the aid of computationally assisted symmetric docking technique (Boyken et al., 2016). Baker et al. individually designed cage-like protein nanomaterials with either dodecameric tetrahedral (T) symmetry or tetraicosameric octahedral (O) symmetry by using homo *C*_3_ symmetrical AcpS protein as building blocks (King et al., 2012). Meanwhile, after docking and further reconstructing trimeric KDPG aldolase at the vertex of decahedron, the variant expressed in *E. coli.* could directly assemble into a uniform hyperstable 60-meric protein icosahedron with 25 nm outer diameter (Hsia et al., 2016). Nay, this strategy can be also employed to design more sophisticated, hyperfine structures through co-assembly of different proteins. Baker et al. designed new protein cage based on icosahedron geometry with *C*_5_-symmetric pentamer at the vertex and *C*_3_-symmetric trimer on the surface. Interestingly, after gene recombination and protein expression in *E. coli.*, 12 pentamers and 20 trimers spontaneously aggregated to get ordered rhombic triacontahedron with 120 subunits and 40 nm in outer diameter, which is comparable to those of native viral capsids (Bale et al., 2016). Meanwhile, if docking *C*_5_-symmetric pentamer at the vertex and *C*_2_-symmetric dimer on the edge, or *C*_3_-symmetric trimer at the vertex and *C*_2_-symmetric dimer on the edge, both of them could be designed for icosahedron and dodecahedron respectively with atomic level accuracy.

Besides, reconstructing proteins with amphiphilic interfaces can be also employed to design protein superstructures *via* amphiphilic self-assembly. Huang and Mann covalently conjugated hydrophobic PNIPAAm polymer on hydrophilic BSA protein, which could further arrange on water droplet/oil interface to form a single protein-layered “proteinosome” with 20–50 μm diameter (Huang et al., 2013). Yan et al. fused a hydrophobic polypeptide tail onto allosteric adenylate kinase (AKe) surface, which could spontaneously assemble into protein nanofilaments. Due to the ligand mediated allosteric effect of AKe, specific Ap5A ligand could modulate the assembling architectures being transformed between 1D nanofilament and 2D crystalline nanosheet through AKe conformation folding and unfolding (Xu et al., 2019).

## Biofunctionalization of Protein Superstructures

Protein self-assembly through rationally designed supramolecular interfaces has proved the potential access to construct various sophisticated protein nanostructures. It has been acknowledged that protein nanostructures afforded quite excellent properties like structural stability and recyclability than protein itself. Therefore, protein assemblies can be used as templates to develop functional biomaterials and might exhibit great potential in biomimetic materials, biomedical diagnosis and therapy, and so on. Scientists can mediate the self-assembly of natural functional proteins, including enzymes (Gao et al., 2014), fluorescent proteins (Li Z. et al., 2019), etc., into advanced functional nanomaterials not only with intrinsic functions of protein building blocks, but also afforded new features. However, most proteins don’t show any biological functions. How to functionalization of protein assemblies is turned to be one of the most critical scientific issues up to now. The key strategy is introducing proper functional units by the aid of protein properties and assembly structures to get the better performance than protein itself. Here we just shortly discussed few strategies for making functional protein nanomaterials, such as encapsulation of functional cargoes inside superstructures, adsorption of functional units on protein assemblies, and reconstruction of functional centers on protein building blocks.

### Encapsulation

Some protein nanostructures, such as polyhedral cages, nanotubes and capsules, afforded hollow structures, which can be employed to physically encapsulate some functional cargoes, including drugs, enzymes, functional DNA/RNA, fluorescent probes and so on. Chakraborti et al. (2019) filled active enzyme (lysozyme) into FT cages to make three-dimensional protein arrays, which exhibited an additional chaperone-like effect with increasing both thermostability and enzymatic activity of the encapsulated enzyme. GroEL nanotubes was also used by Aida et al. to encapsulate protein models (GFP), which might be applicable for ATP-responsive intracellular delivery (Biswas et al., 2013). Besides, “giant proteinosome” could be used to encapsulate hundreds of components for cell-free gene expression system (Huang et al., 2013), in cellular enzymatic reaction (Huang et al., 2014) and programmed cargo release (Liu et al., 2016). Uneo et al. used self-assembled porous protein crystals for molecular recognition and storage of exogenous substances in living cell as well as applicable for bioorthogonal chemistry (Abe et al., 2017).

### Adsorption

Protein intrinsic charge distribution or recognition affinity makes it possible for further adsorb some functional cargoes even after self-assembly. For example, avidin-mediated CCMV crystals could be further functionalized with enzymes to develop recyclable nanoenzymes through avidin recognition with enzyme conjugated biotin (Liljestrom et al., 2014). Liu et al. constructed a photo controlled reversible microtubule assembly system through adsorbing β-cyclodextrin (β-CD) and photochromic arylazopyrazole (AAP) on the surface. The host-guest interaction between β-CD and AAP could be switched by light to reversibly controlled the intertubular aggregation behaviors of microtubules, which might be employed for the treatment of diseases related to improper protein aggregations (Zhang et al., 2018). Electrostatic interaction is also an effective strategy to immobilize functional units on protein superstructures. electrostatic co-crystallization of a cationic phthalocyanine (Pc) and negatively charged TMV was constructed by Torre and Kostiainen to prepare exceptionally long fibers, which could behavior as heterogeneous catalysts for continuous flow photo-oxidation processes (Anaya-Plaza et al., 2019). Two dimensional SP1 superlattices could further electrostatically capture size-matched QDs to construct artificial chloroplast mimics (Zhao et al., 2017).

Moreover, the protein nanostructures can be also used for constructing more complicated macro protein-inorganic composites. Wang et al. developed 2D periodic lattices *via* self-assembly of cylinder-shaped TMV disks, such structures can be used to modulate the organization of inorganic nanoparticles (AuNPs or QDs) into highly organized nanostructures from 0D to 2D sophisticated patterns (Zhang et al., 2019). Mezzenga et al. used β-lactoglobulin (BLG) amyloid fibrils as templates for ultralight BLG-CaCO_3_ (Shen et al., 2017) and BLG-AuNP hybrid aerogels (Nystrom et al., 2018). These specific hybrids materials might be the suitable candidates for catalysis, sensing, and environmental or biomedical applications.

### Reconstruction

Protein reconstruction is thought to be the most effective method for functionalization of protein nanomaterials for the fact that functional fragment can be directly introduced into protein structures. Scientists usually used chemical modification or biological genetic recombination to construct functional protein nanomaterials. Francis et al. site-specifically modified TMV coat proteins with chromophore pairs, and found TMV disks could not only prevent the aggregation-induced quench of chromophores but also greatly improved FRET transfer efficacy (Delor et al., 2018). If introducing GPx catalytic centers on TMV *via* site-directed gene mutagenesis, the reengineered nanostructures showed ultrahigh catalytic activity and stability (Hou et al., 2012). Liu et al. installed GPx centers to SP1 surface while SOD mimics on dendrimers, the co-assembled nanowires afforded excellent ability in resisting oxidation damage of organisms (Sun et al., 2015). If rationally arranging chromophores on the similar structures, the protein nanomaterials could be designed to construct artificial light harvesting systems (Sun et al., 2016a). Besides, other kinds of functional materials have been developed, such as viral capsid grafting for targeted imaging/drug delivery (Brauer et al., 2019) and nanoreactors (Jordan et al., 2016), artificial metalloenzymes construction on protein structures (Ueno et al., 2013), artificial GPx nanoenzyme on GST nanowires (Hou et al., 2013), and others.

## Conclusion and Future Perspectives

To date, protein self-assembly through designed supramolecular interfaces has been widely broadened and has been proved to be a powerful tool to design protein nanostructures. It is fair to say that the great diversity and versatility of various protein structures and interfacial interactions provide infinite possibilities for hierarchical protein structures and potential biological applications. However, since protein self-assembly is still a new emerging area, in our opinion, three following directions should be attended in the future: (1) *In vivo* synthesized protein superstructures. That is, directly synthesizing targeted protein superstructures in sophisticated, condensed cellular media through the transformation of protein sequence. (2) Disease-related protein assembly/disassembly. The essence of protein self-assembly should be returned to the acknowledgment of biological assembly behaviors like Alzheimer’s disease, transmissible spongiform encephalopathy (TSE), Parkinson’s disease, etc. The study on protein assembly might be helpful to reverse the pathogenic process. (3) Advanced functional protein materials. Protein assembly has showed great priority in constructing functional protein nanomaterials. These materials would be potentially applicable for implantable biomaterials, disease treatment and industrial productions in coming days. We hope this tutorial review would provide a rapid survey for assembly with designed protein interfaces and functionalization of protein superstructures, and might be helpful to accelerate the pace of further discoveries.

## Author Contributions

HS and JL conceived the idea and organized this mini review. All authors contributed to writing, editing, and literature review, and approved the contents for publication.

## Conflict of Interest

The authors declare that the research was conducted in the absence of any commercial or financial relationships that could be construed as a potential conflict of interest.
